# ONQUADRO: a database of experimentally determined quadruplex structures

**DOI:** 10.1093/nar/gkab1118

**Published:** 2021-11-19

**Authors:** Tomasz Zok, Natalia Kraszewska, Joanna Miskiewicz, Paulina Pielacinska, Michal Zurkowski, Marta Szachniuk

**Affiliations:** Institute of Computing Science, Poznan University of Technology, 60-965 Poznan, Poland; Institute of Computing Science, Poznan University of Technology, 60-965 Poznan, Poland; Institute of Computing Science, Poznan University of Technology, 60-965 Poznan, Poland; Institute of Computing Science, Poznan University of Technology, 60-965 Poznan, Poland; Institute of Computing Science, Poznan University of Technology, 60-965 Poznan, Poland; Institute of Computing Science, Poznan University of Technology, 60-965 Poznan, Poland; Institute of Bioorganic Chemistry, Polish Academy of Sciences, 61-704 Poznan, Poland

## Abstract

ONQUADRO is an advanced database system that supports the study of the structures of canonical and non-canonical quadruplexes. It combines a relational database that collects comprehensive information on tetrads, quadruplexes, and G4-helices; programs to compute structure parameters and visualise the data; scripts for statistical analysis; automatic updates and newsletter modules; and a web application that provides a user interface. The database is a self-updating resource, with new information arriving once a week. The preliminary data are downloaded from the Protein Data Bank, processed, annotated, and completed. As of August 2021, ONQUADRO contains 1,661 tetrads, 518 quadruplexes, and 30 G4-helices found in 467 experimentally determined 3D structures of nucleic acids. Users can view and download their description: sequence, secondary structure (dot-bracket, classical diagram, arc diagram), tertiary structure (ball-and-stick, surface or vdw-ball model, layer diagram), planarity, twist, rise, chi angle (value and type), loop characteristics, strand directionality, metal ions, ONZ, and Webba da Silva classification (the latter by loop topology and tetrad combination), origin structure ID, assembly ID, experimental method, and molecule type. The database is freely available at https://onquadro.cs.put.poznan.pl/. It can be used on both desktop computers and mobile devices.

## INTRODUCTION

G-quadruplexes (G4s) are unique structures folded in G-rich nucleic acids (1,2), found in eukaryotic, prokaryotic, and viral genomes (1,3,4). In biological processes, they play crucial regulatory roles by participating in telomere maintenance, the regulation of gene expression, DNA replication, etc. (2,3,5,6). A recent hypothesis, coined as the quadruplex world, suggests that G4s may have been the molecules to initiate life on Earth (7). It takes its cue from the ability of guanines to form stable G-tetrads, the basic building units of a quadruplex. In a tetrad, four guanines arranged in the same plane connect via hydrogen bonds such that each acts as a donor of two hydrogen bonds at the Watson-Crick edge and an acceptor at the Hoogsteen edge (1,8,9). A complete G4 is assembled from at least two G-tetrads stacked one above another, and is stabilised by monovalent cations located in the ion channel (5,9). The stacking interactions of G-tetrads occurring independently of backbone connectivity define a G4-helix (10).

The general definition of what constitutes a quadruplex does not capture the complexity of its structure and the diversity of its features (2,8,9,11–14). Meanwhile, the latter is subjected to various studies, aiming - among others - to associate the motif’s conformation with its function, find the relationship between its sequence and the higher-level structure, cluster and classify quadruplexes, learn about and fully describe their properties. We already know that tetrads can form from guanine as well as non-guanine nucleotides (15). Their spatial arrangement to their stacking neighbours is diverse, defined by the rise and twist parameters. The topologies of secondary structures differ in both the tetrad and the quadruplex set, as reflected in the ONZ classification (16). They are influenced by the number of strands contributing to the motif, their lengths, and directionality. Strands may form loops, which can be a part of the quadruplex - cf. Webba da Silva formalism (17). The list of analysed attributes also includes the glycosidic bond angles, the groove width, the number of stacked tetrads, or G-tract continuity, and is probably not yet complete (11).

In the past decade, the unique structure of the quadruplex has focused the attention of many researchers, especially in medical sciences. G4s have become therapeutic targets, that is, for cancer and antiviral treatment (18–20). In the latter case, increased interest in targeting G-quadruplexes in viral genomes was prompted by the COVID-19 pandemic. The frequency and localisation of putative quadruplex sequences in different viral taxa, G4-binding viral domains, and the potential of G4s as viral biosensors were investigated (20–25). These and other quadruplex studies provided a wealth of data for collection, organisation, and further analysis (26–28). It has initiated the development of computational methods and bioinformatics tools dedicated to G4s. Most of these deal with sequence data storage and processing (15,29–35). A few address higher-level structures (10,36–39), including databases that store G4-related data (36,40,41) - none, however, collects complete information about quadruplex structures at all levels of their organisation.

ONQUADRO is a new comprehensive database system that collects and shares data on tetrads, quadruplexes, and G4-helices, whose three-dimensional structures have been determined experimentally. Baseline data are regularly downloaded from the Protein Data Bank (42) and supplemented with parameters computed by specialised procedures of the system’s engine. The incorporated programs prepare visualisations of the secondary and tertiary structure models of each motif. The analytical module generates statistics of the distribution of the structural parameters in the set of tetrads and quadruplexes. The system allows users to subscribe to a newsletter about all database newcomers. ONQUADRO, designed for use on desktop computers and mobile devices, is freely available at https://onquadro.cs.put.poznan.pl/.

## METHOD OUTLINE

**Figure 1.**
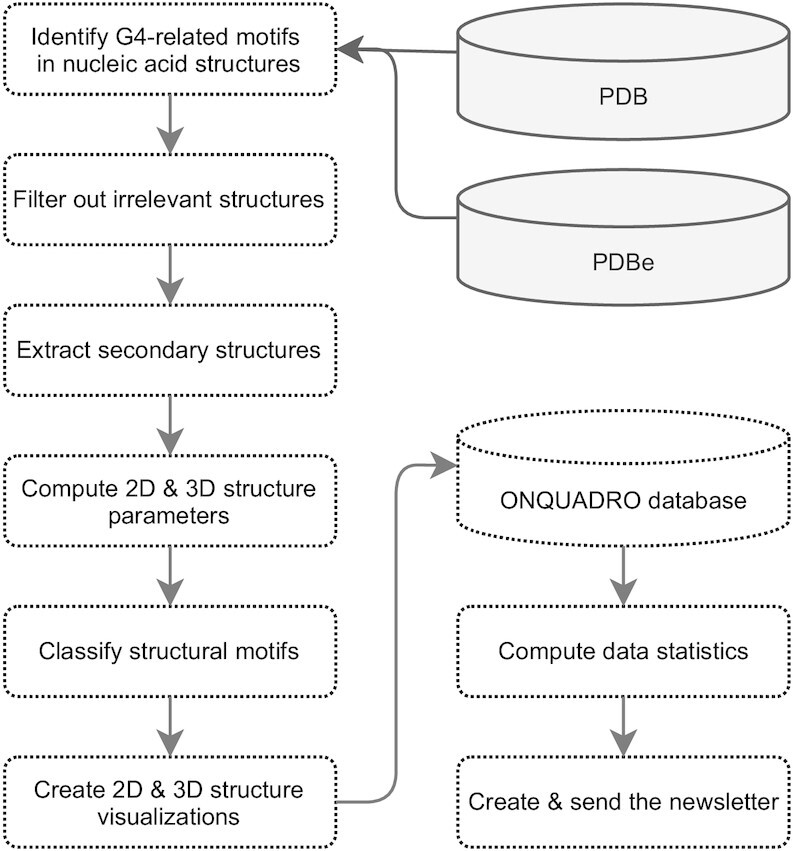
Data flow during the weekly ONQUADRO update.

Every Thursday, the update module of ONQUADRO connects to the PDB FTP site and searches for new information about nucleic acids (including protein-nucleic acid complexes). Next, it queries PDBe (43) for biological assemblies to associate them with the items found. The module creates a list with identifiers of newly added, modified, or deleted structures containing tetrads, quadruplexes, and G4-helices. Changes to the ONQUADRO database are made from the list of modified and deleted structures; entries for new motifs are created and added. The process of new data preparation takes place in several steps (Figure 1).

For each quadruplex, it derives the secondary structure and prepares its representation in two-line extended dot-bracket notation, computes the rise and twist parameters, identifies the number of contributing strands and tetrads, determines the strand direction, finds loops to calculate their lengths and types, classifies it according to Webba da Silva formalism based on its loop topology and tetrad combination (17), and assigns an ONZ class from the secondary structure topology (16). If the nucleic acid contains metal ions, the procedure determines their position relative to the quadruplex. Then, it describes every tetrad by planarity, chi angle value and type, ONZ class, and tetrad combination. In the next step, graphical models of the secondary and tertiary structure of every motif are prepared. These include a classical diagram, arc diagram, layer diagram, and 3D molecule models. After calculating all the parameters and preparing graphical models, the system populates the database and maintains the relationships between the entries.

Once the database is updated, the statistical analysis module generates graphs and tables of the data distribution. Statistics available for G4s are (i) the number of quadruplexes as a function of the number of constituent tetrads; (ii) the abundance of the set of uni-, bi-, and tetramolecular quadruplexes; (iii) ONZ class coverage by uni-, bi-, and tetramolecular quadruplexes; (iv) the geometric class distribution based on glycosidic bond angles and loop topology; (v) the loop length distribution in the subsets of lateral, propeller and diagonal loops and (vi) the distribution of the twist and rise values. Statistics prepared for tetrads include: (i) the distribution of tetrads concerning their sequence and molecule type; (ii) the coverage of ONZ classes by uni-, bi-, and tetramolecular tetrads; (iii) the chi angle value distribution in the ONZ classes; (iv) ONZ class coverage by ions and (v) the planarity value distribution in the tetrad set.

Finally, the system creates a hypertext newsletter listing all changes to the database and sends it to subscribers.

## IMPLEMENTATION

The ONQUADRO system consists of the database, web application, and computational engine. It runs on a quad-core machine with 8GB RAM in a Ubuntu GNU/Linux environment, hosted and maintained by the Institute of Computing Science, Poznan University of Technology.

### Database

ONQUADRO has been developed as a relational database in PostgreSQL. It is composed of tables that correspond to PDB structures, G4-helices, quadruplexes, tetrads, tetrad pairs, base pairs, nucleotides, tracts, loops, and ions. The database stores the following information about every nucleic acid structure: PDB ID, assembly ID, experimental method, resolution, deposition, release and revision dates, molecule name, a secondary structure diagram, and a 3D structure. The secondary and tertiary structures are collected separately for tetrads, quadruplexes, and G4-helices. Additionally, the database contains data on the ONZ class, type (uni-, bi-, or tetramolecular), loops, and ions for quadruplexes, ONZ class and planarity for tetrads, strand direction, rise and twist for tetrad pairs, stericity and edge names for base pairs, model, chain, and glycosidic bond for nucleotides.

All sequences in ONQUADRO are coded in a one-letter format, in the 5′–3′ direction. The secondary structures of tetrads, quadruplexes, and G4-helices are represented using dot-bracket notation - an unpaired nucleotide corresponds to a dot and a base pair to a pair of opening and closing brackets. Since in the considered motifs, each nucleotide pairs with two others, basic dot-bracket notation is not sufficient to unambiguously encode the secondary structure of a tetrad, so for a quadruplex or G4-helix. Therefore, in (16), we introduced a two-line dot-bracket and used an extended set of brackets to label paired nucleotides. This includes parentheses ( ), square brackets [ ], curly brackets { } and angle brackets 〈 〉. An arc diagram representing the secondary structure is also adjusted to unambiguously reflect all pairings. It is associated with dot-bracket notation - the top of the diagram corresponds to the first line of the dot-bracket notation, and the bottom part corresponds to the second line (16). The secondary structure of every motif is also visualised in a classical diagram. The 3D structure is represented by a layer diagram and three molecular models (ball-and-stick, surface, vdw-balls).

### Computational engine

**Figure 2.**
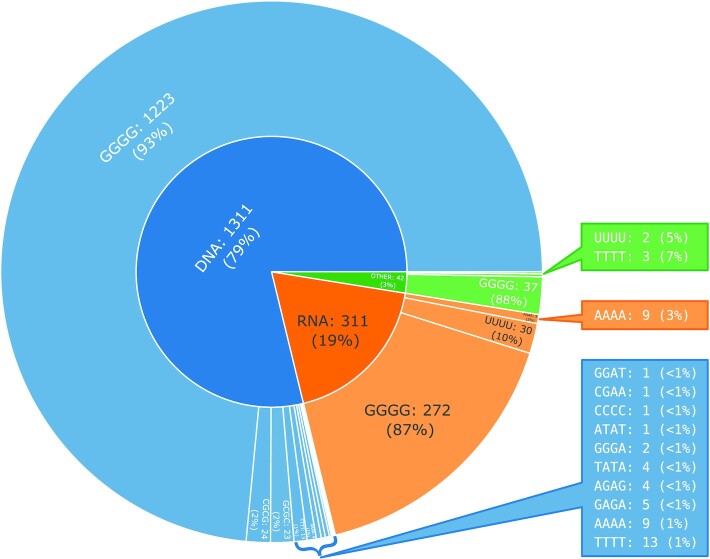
Example statistics generated by ONQUADRO: quadruplexes by the number of constituent tetrads.

The computational engine is composed of scripts utilising in-house and third-party procedures, responsible for data collection, quadruplex identification, computation of structure parameters, secondary structure annotation, visualisation of the secondary and tertiary structure models, database queries, generation of statistics, and newsletter preparation. DSSR (--pair-only mode) (36) and ElTetrado (39) functionalities are applied to identify quadruplexes, tetrads, and G4-helices in nucleic acid structures. Procedures from ElTetrado (39) and the BioCommons library (44) compute a variety of structure parameters. The VARNA-based routine (45) creates a classical diagram of the secondary structure. The R-Chie-driven function (46) produces a top-down arc diagram. The embedded LiteMol (47) module generates models (ball-and-stick, surface, vdw-balls) of the three-dimensional structure. The Python script draws a layer diagram of the quadruplex based on the data in JSON format obtained from ElTetrado. Every nucleotide in the diagram is colour-coded – yellow indicates the anti conformation, orange is for syn. The script optimises the quadruplex position in three-dimensional space to get a clear view of the G4, with the least number of crossing strands. The optimisation algorithm has been implemented in C++. Statistics are generated using the script in R and the Plotly library. They self-update whenever new entries appear in the database (Figure 2).

### Web application

The web application provides an interface for the ONQUADRO system. The client application has been created using the Angular framework and Bootstrap styling sheet; the server is implemented in C#. The client and the server communicate via REST API.

**Figure 3.**
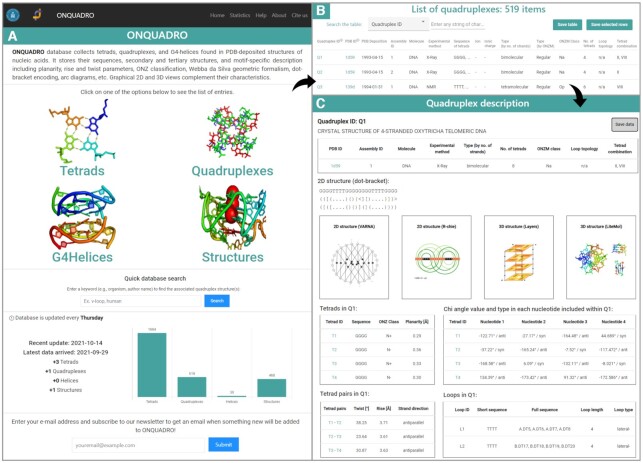
ONQUADRO interface: (**A**) main page, (**B**) quadruplex table and (**C**) quadruplex details based on 1D59 structure.

Figure 3 presents screenshots of the ONQUADRO system. On the homepage (https://onquadro.cs.put.poznan.pl/), users can see brief information about the database resources and the latest update. From this page, users can access the sets of tetrads, quadruplexes, G4-helices or structures. The selected subpage displays a list of items with a basic description. Some data are clickable - they allow detailed structural information to be viewed about the selected element or link to the corresponding page in the Protein Data Bank. The table can be sorted (ascending or descending) for the contents of any column by clicking on the column header. Users can search the list of items by using the *Search the table* option and typing the string of interest. Searching runs in real-time. The element counter at the top of the page shows how many elements contain the queried string. These elements are displayed in the table. Users can save the content of each table as a whole (*Save table* button) or a selected part (*Save selected rows* button after clicking on the check-boxes in the rightmost column). Tabular data (from any subpage) are downloaded in a CSV file, structure visualisations can be saved in SVG format.

The *Statistics* option in the menu bar leads to a page listing statistics for tetrads and quadruplexes. User-selected stats are displayed in graphical (pie chart, tree map, or bar plot) and tabular form. Plots can be saved in HTML format. They are interactive - upon clicking the plot, users can enlarge fragments and see selected parts of the data.

## CONCLUSIONS

ONQUADRO gathers information about all tetrads, quadruplexes, and G4-helices found in experimentally determined nucleic acid structures deposited in the Protein Data Bank (42). The system’s computational engine combines self-developed procedures to annotate these motifs, derive their secondary structures, classify them according to geometric formalism (17) and topological ONZ nomenclature (16), represent the secondary structure in dot-bracket notation and a specially adjusted top-down arc diagram, draw a 3D model in a schematic layer diagram, and trigger statistics. Some are G4-adapted routines applied in our previously released tools; others are brand new and have not yet been published (e.g. automatic creation of layer diagrams - a much-needed function in the research community). The user-friendly interface allows browsing of the database contents divided into four subsets (tetrads, quadruplexes, G4-helices, PDB structures), searching and sorting the data by various parameters and keywords, displaying and downloading detailed structural information on selected motifs, viewing and downloading statistics in graphical and textual form. ONQUADRO is a unique online resource that takes a comprehensive approach to collecting and sharing quadruplex information. We hope it will facilitate the study of G4 structures and their modelling *in silico* - a great challenge for modern structural bioinformatics.

## DATA AVAILABILITY

ONQUADRO is a continuously maintained, weekly self-updating resource available at https://onquadro.cs.put.poznan.pl. No registration or login is required to access the data and take full advantage of the system’s functionality.
